# Nova score for the consumption of ultra-processed foods: description and performance evaluation in Brazil

**DOI:** 10.11606/s1518-8787.2021055003588

**Published:** 2021-04-05

**Authors:** Caroline dos Santos Costa, Franciane Rocha de Faria, Kamila Tiemann Gabe, Isabela Fleury Sattamini, Neha Khandpur, Fernanda Helena Marrocos Leite, Eurídice Martínez Steele, Maria Laura da Costa Louzada, Renata Bertazzi Levy, Carlos Augusto Monteiro

**Affiliations:** I Universidade de São Paulo Núcleo de Pesquisas Epidemiológicas em Nutrição e Saúde São PauloSP Brasil Universidade de São Paulo. Núcleo de Pesquisas Epidemiológicas em Nutrição e Saúde. São Paulo, SP, Brasil; II Universidade de São Paulo Faculdade de Saúde Pública Programa de Pós-Graduação em Nutrição em Saúde Pública São PauloSP Brasil Universidade de São Paulo. Faculdade de Saúde Pública. Programa de Pós-Graduação em Nutrição em Saúde Pública. São Paulo, SP, Brasil; III Universidade Federal de Rondonópolis Instituto de Ciências Exatas e Naturais RondonópolisMT Brasil Universidade Federal de Rondonópolis. Instituto de Ciências Exatas e Naturais. Rondonópolis, MT, Brasil; IV Universidade de São Paulo Faculdade de Saúde Pública Departamento de Nutrição São PauloSP Brasil Universidade de São Paulo. Faculdade de Saúde Pública. Departamento de Nutrição. São Paulo, SP, Brasil; V Universidade de São Paulo Faculdade de Saúde Pública Programa de Pós-Graduação em Saúde Global e Sustentabilidade São PauloSP Brasil Universidade de São Paulo. Faculdade de Saúde Pública. Programa de Pós-Graduação em Saúde Global e Sustentabilidade. São Paulo, SP, Brasil; VI Universidade de São Paulo Faculdade de Medicina Departamento de Medicina Preventiva São PauloSP Brasil Universidade de São Paulo. Faculdade de Medicina. Departamento de Medicina Preventiva. São Paulo, SP, Brasil

**Keywords:** Food Consumption, Ultra-processed Food, Diet Surveys, methods, Surveys and Questionnaires, Validation Study

## Abstract

**OBJECTIVE:**

To describe the Nova score for the consumption of ultra-processed foods (UPF) and evaluate its potential in reflecting the dietary share of UPF in Brazil.

**METHODS:**

This study was conducted in São Paulo with a convenience sample of 300 adults. Using a tablet, participants answered a 3-minute electronic self-report questionnaire on the consumption of 23 subgroups of UPF commonly consumed in Brazil, regarding the day prior the survey. Each participant score corresponded to the number of subgroups reported. The dietary share of UPF on the day prior to the survey, expressed as a percentage of total energy intake, was calculated based on data collected on a 30-minute complete 24-hour dietary recall administered by trained nutritionists. The association between the score and the dietary share of UPF was evaluated using linear regression models. The Pabak index was used to assess the agreement in participants’ classification according to the fifths of Nova score and the fifths of dietary share of UPF.

**RESULTS:**

The average dietary share of UPF increased linearly and significantly with the increase of the Nova score for the consumption of ultra-processed foods. We found a substantial agreement in participants’ classification according to the fifths of the distribution of scores and the fifths of the dietary share of UPF (Pabak index = 0.67). Age was inversely associated with a relatively high frequency of UPF consumption (upper fifth of the distribution) for both score and dietary share of UPF.

**CONCLUSION:**

The Nova score for the consumption of ultra-processed foods, obtained in a quick and practical manner, shows a good potential in reflecting the dietary share of UPF in Brazil.

## INTRODUCTION

According to the Nova food classification system^1^, ultra-processed foods (UPF) are industrial formulations of food-derived substances that contain little or no whole food, often including flavorings, colorings, emulsifiers, and other cosmetic additives to provide them palatability or even hyperpalatability. Most ingredients and processes used to manufacture these foods are exclusively used by the food industry^2^.

Nutritional surveys conducted with probabilistic samples from the population of several countries show that UPF intake, measured by the percentage of total energy intake related to these foods, is strongly and inversely related to the nutritional quality of the diet^3^. Systematic reviews of well-conducted and large cohort studies show that the percentage of total energy intake from UPF is directly associated with the risk of chronic non-communicable diseases such as obesity, diabetes, hypertension, dyslipidemias, cardiovascular and cerebrovascular diseases, cancer in general, breast cancer, and depression, as well as with premature deaths from any cause^4^.

Studies addressing the association between UPF consumption and nutritional quality of the diet or risk of chronic diseases measured UPF dietary contribution using data-collection tools that require experienced interviewers and time and disposition from interviewees, as 24-hour dietary recalls; or tools that require participants with high education level, time, and disposition, as in food records or food frequency questionnaires^11^. Given the complexity of these data-collection instruments, the intake of ultra-processed foods in many populations is still unknown and, even more, difficult to be monitored, thus hampering the formulation and evaluation of public policies aimed at reducing UPF consumption.

To monitor the consumption of UPF by the Brazilian adult population, and as part of the Surveillance System for Risk and Protective Factors for Chronic Diseases by Telephone Survey (VIGITEL), authors of this article developed a simplified instrument addressing questions on the previous-day dietary intake of a list of 13 subgroups of ultra-processed foods (answered with “yes” or “no”). Part of the VIGITEL’s annual questionnaire since 2018^12^, the instrument enables the calculation of a score of UPF consumption ranging from zero to thirteen^13^ – equivalent to the number of subgroups consumed in the previous day by the interviewees. A study conducted with a convenience sample of 150 participants showed a good agreement between the score and the dietary share of UPF^14^.

As part of the development of the NutriNet Brasil cohort study, we built a second simplified instrument to evaluate UPF consumption that waives interviewers’ participation, conducted in mobile phones, tablets, or computers^15^ – the Nova screener for the consumption of ultra-processed foods. This instrument provides the Nova score for the consumption of ultra-processed foods, whose description and ability to reflect the dietary share of UPF in Brazil are presented below.

## METHODS

### Sample

This study was conducted in the city of São Paulo with a convenience sample of 300 adults aged 18 years or older, users of two health centers of the Universidade de São Paulo (USP) and employees and students at USP.

### Data Collection

Two nutritionists trained by one of the authors of this article (CSC) collected the study data between September and November 2019. All participants were informed about the study purpose and invited to participate. After agreeing to participate by signing the consent form, the participants informed their gender, age, and education level. Then, using a tablet and without the nutritionist aid, participants answered to the Nova screener for the consumption of UPF, checking all items within a list that had been consumed the day before (checkbox format). The average time spent to complete the answers was three minutes. After completion, the nutritionist conducted a 24-hour dietary recall (24-hR), where participants informed all foods and the amount they had consumed the day before. The dietary recall took on average 30 minutes.

### Nova screener for the consumption of ultra-processed foods

**Figure 1:**
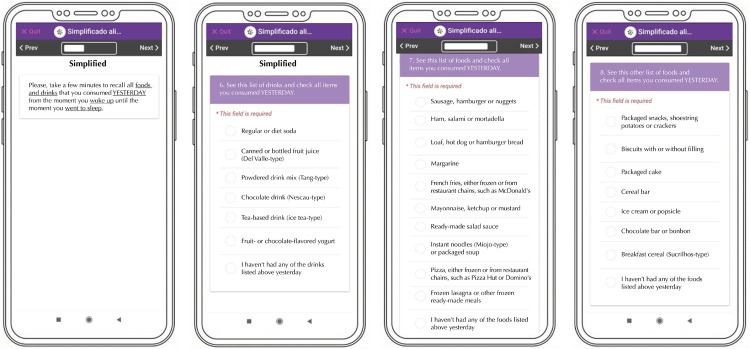
Nova screener for the consumption of ultra-processed foods on the Epicollect5 Data Collection® platform.

As in the instrument employed by the VIGITEL system, the Nova screener for the consumption of ultra-processed foods was developed to include UPF subgroups with greater participation in the daily energy intake, estimated by the national food consumption survey conducted by the 2008–2009 Household Budget Survey(POF) of the Brazilian Institute of Geography and Statistics (IBGE)^16^. After unfolding some of the 13 subgroups of the original instrument, the Nova screener presents a list of 23 subgroups of UPF. The questions addressing the intake of each of these subgroups are presented on three categories: beverages (six subgroups); products that replace or accompany meals (ten subgroups); and products often consumed as snacks (seven subgroups), as shown in Figure 1. Questions were uploaded into the tablet with the Epicollect5 Data Collection^a^ software, which stores participants’ answers as a database.

### 24-hour Dietary Recall (24-hR)

The 24-hR was applied by the nutritionists, using the five-stage multiple-pass method^17^. First, participants report, quick and uninterruptedly, all foods and beverages consumed. Then, the nutritionist asks for other foods or beverages that the interviewee might have forgotten to report, based on a list. The participant is then queried about the type, time, and place of each meal, followed by the provision of details such as preparation mode, origin, quantities, homemade measures and sizes, as well as other foods addition (e.g., sugar). To finalize, the interviewer lists the entire report to the interviewee, reviewing and stimulating the report of foods possibly forgotten and/or omitted.

This study was approved by the Research Ethics Committee of the School of Public Health of the Universidade de São Paulo (CAAE: 83221317.5.0000.5421; opinion no.: 2.517.894) and all participants signed the informed consent form.

### Data Analysis

The Nova score of each participant was calculated from the sum of UPF subgroups reported among the 23 listed, thus ranging from 0 to 23. To estimate the dietary share of UPF in the previous-day food consumption, each item reported in the 24-hR was initially classified into ultra-processed or non-ultra-processed, according to the Nova classification^1,2^. Then, the consumed quantity of each item, reported in homemade measures, was transformed into grams and converted into calories using the Composition Table of Foods Consumed in Brazil^18^. Finally, we calculated the total calories consumed, the calories from UPF, and the percentage of total calories from UPF.

To assess the association between the Nova score and the dietary share of UPF, we examined the variation in the average percentage of calories from UPF according to the score variation, expressed continuously and also at intervals corresponding to fifths of its distribution. In both cases, linear regression models were used to test the linear trend. Then, the degree of agreement in participants’ classification according to the fifths of the distribution of the percentage of calories from UPF and the fifths of the distribution of the Nova score was evaluated by calculating the prevalence-adjusted and bias-adjusted kappa (Pabak) index^19^. Values greater than 0.80 indicate an almost perfect agreement; between 0.61 and 0.80, a substantial agreement; between 0.41 and 0.60, moderate; between 0.21 and 0.40, fair; and equal to or less than 0.20, slight^20^. We also compared the variation in the prevalence of relatively high consumption of UPF according to age group, determined by two alternative criteria: consumption equivalent to that observed in the upper fifth of the distribution of Nova score; and consumption equivalent to that observed in the upper fifth of the distribution of the percentage of calories from UPF.

The analyses were performed using the Stata^®^ 16.1 software, and the Pabak index was calculated using the WINPEPI (PEPI-for-Windows) 11.65 software^b^.

## RESULTS

Among the 300 volunteers interviewed, most were female (71.3%), aged between 25 and 59 years (72.3%), who completed secondary education, or completed or are attending tertiary education (80.7%), as shown in Table 1.

**Table 1:** Distribution according to sociodemographic variables of adult users of health centers and employees and students at the Universidade de São Paulo (n = 300). São Paulo, Brazil, 2019.

Variables	n	%
Gender		
Male	86	28,7
Female	214	71,3
Age (years)		
18–24	34	11,4
25–34	79	26,3
35–44	71	23,7
45–59	67	22,3
60+	49	16,3
Education level		
Some secondary education	58	19,3
Secondary education	114	38,0
Some tertiary education or tertiary education	128	42,7

Table 2 describes the consumption frequency of each subgroup included in the Nova screener for the consumption of UPF on the day prior to the interview. About one in every three participants reported having consumed margarine (38.0%), loaf, hot dogs, or hamburger bread (33.0%), and regular or diet soda (30.7%). Between 15% and 20% reported having consumed biscuits with or without filling (19.7%), packaged snacks, shoestring potatoes or crackers (16.3%), and chocolate bar or bonbon (15.0%). Less than 15% of the interviewees reported consuming food from the other subgroups on the day prior to the interview.

**Table 2:** Consumption frequency (%) of foods included in the Nova screener for the consumption of ultra-processed foods on the day prior the interview. Adult users of health centers and employees and students at the Universidade de São Paulo (n = 300). São Paulo, Brazil, 2019.

Foods	%
Margarine	38.0
Loaf, hot dog, or hamburger bread	33.0
Regular or diet soda	30.7
Biscuits with or without filling	19.7
Packaged snacks, shoestring potatoes or crackers	16.3
Chocolate bar or bonbon	15.0
Ham, salami or mortadella	14.7
Sausage, hamburger or nuggets	13.3
Fruit- or chocolate-flavored yogurt	12.7
Canned or bottled fruit juice (Del Valle-type)	12.7
Powdered drink mix (Tang-type)	12.0
Mayonnaise, ketchup or mustard	11.7
Ice cream or popsicle	10.3
Chocolate drink (Nescau-type)	8.3
French fries, either frozen or from restaurant chains such as McDonald’s	5.3
Instant noodles (Miojo-type) or packaged soup	5.3
Tea-based beverage (ice tea-type)	4.0
Pizza, either frozen or from restaurant chains, such as Pizza Hut or Domino’s	3.7
Frozen lasagna or other frozen ready-made meals	3.3
Ready-made salad sauce	3.0
Packaged cake	2.7
Cereal bar	2.7
Breakfast cereal (Sucrilhos-type)	1.7

Table 3 describes the distribution of the Nova score for the consumption of UPF, which is equivalent to the number of subgroups consumed on the day before the interview. Scores ranged from 0 to 9, but 1 (19.7%), 2 (20.3%), 3 (19.3%), and 4 (14.0%) were the most common; 9.0% of participants reached null scores and 17.7% equal to or higher than 5. As shown in Table 3, the average percentage of dietary share of UPF, calculated based on the 24-hour dietary recall, increases linearly and significantly with the increase in the UPF consumption score.

**Table 3:** Dietary share of ultra-processed foods calculated by the 24-hour food recall according to the Nova score. Adult users of health centers and employees and students at the Universidade de São Paulo (n = 300). São Paulo, Brazil, 2019.

Nova score for the consumption of ultra-processed foods	Sample n (%)	Dietary share of ultra-processed foods (% of total energy) Average (95%CI)
0	27 (9.0)	9.4 (2.3–16.6)
1	59 (19.7)	23.8 (19.0–28.6)
2	61 (20.3)	31.6 (26.9–36.4)
3	58 (19.3)	31.1 (26.2–35.9)
4	42 (14.0)	35.6 (29.8–41.3)
5	23 (7.7)	37.5 (29.8–45.2)
6	17 (5.7)	54.1 (45.2–63.1)
7	8 (2.7)	47.0 (34.0–60.1)
8	1 (0.3)	27.5 (-9.4–64.5)
9	4 (1.3)	35.6 (17.1–54.0)^a^
0–1	86 (28.7)	19.3 (15.2–23.4)
2	61 (20.3)	31.6 (26.8–36.5)
3	58 (19.3)	31.1 (26.1–36.1)
4	42 (14.0)	35.6 (29.7–41.4)
5 or +	53 (17.7)	43.9 (38.7–49.1)^a^

95%CI: 95% confidence interval

^a^ P-value for linear trend < 0.001.

Table 4 shows that participants’ distribution, considering their classification based on the fifths of the dietary share of UPF (calculated by the 24-hR) and of the Nova score (0–1, 2, 3, 4 and ≥ 5) indicates substantial agreement between the two criteria (Pabak index of 0.67).

**Table 4:** Distribution (%) according to the fifths of the dietary share of ultra-processed foods and (approximate) fifths of the Nova score for the consumption of ultra-processed foods. Adult users of health centers and employees and students at the Universidade de São Paulo (n = 300). São Paulo, Brazil, 2019.

Fifths of the Nova score for the consumption of ultra-processed foods	Fifths of the dietary share of ultra-processed foods (% of total calories)	0–1	2	3	4	5 or +	Total
Q1 (≤ 11.0)		13.0	2.0	2.3	2.0	0.7	20.0
Q2 (11.1–20.4)		6.0	5.0	4.0	2.7	2.3	20.0
Q3 (20.5–34.8)		4.0	4.7	6.0	2.3	3.0	20.0
Q4 (34.9–49.5)		3.3	5.0	3.7	3.0	5.0	20.0
Q5 (≥ 49.6)		2.3	3.7	3.3	4.0	6.7	20.0
Total		28.6	20.4	19.3	14.0	17.7	100.0

Note: Pabak index (prevalence-adjusted bias-adjusted Kappa) = 0.67.

**Figure 2:**
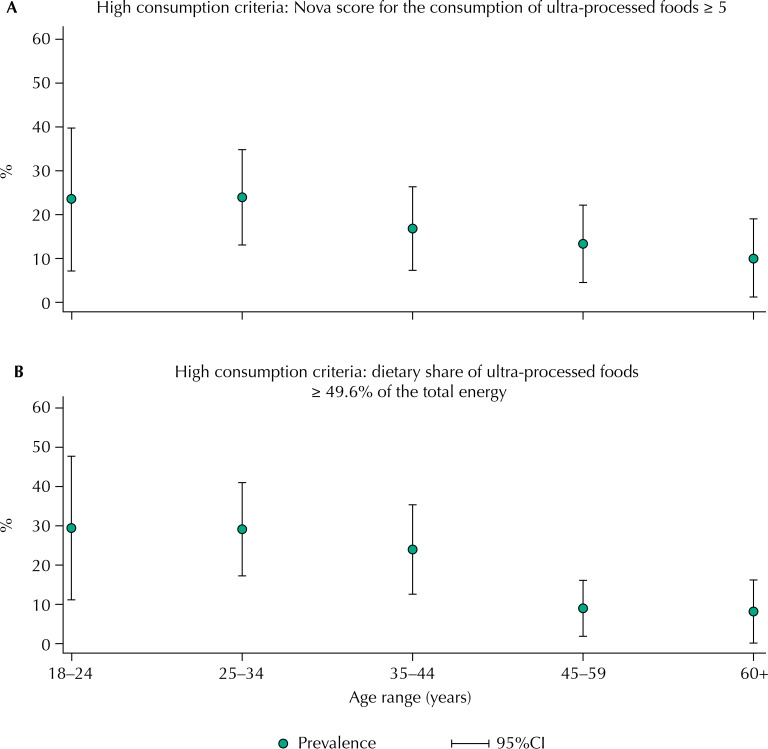
Variation in the prevalence (%) of high consumption of ultra-processed foods according to age group based on two criteria. Adult users of health centers and employees and students at the Universidade de São Paulo (n = 300). São Paulo, Brazil, 2019.

^a^ P-value for linear trend = 0.038.

^b^ P-value for linear trend = 0.001.

Figure 2 shows the variation in the prevalence of relatively high consumption of UPF according to age groups, defined based on the consumption observed, alternatively, in the upper fifth (approximate) of the distribution of the Nova score (≥ 5) and of the distribution of UPF participation in the total caloric intake (≥ 49.6% of the total calories). Using these two criteria, we verified that the prevalence of relatively high consumption of UPF linearly decreases with increasing age (p = 0.038 and p = 0.001, respectively).

## DISCUSSION

Our results indicate that the Nova score for the consumption of ultra-processed foods, obtained with a 3-minute electronic self-report questionnaire, was directly and linearly associated with the percentage of total energy intake from UPF, obtained with a 24-hour dietary recall applied by a trained nutritionist in about 30 minutes. We also found a substantial agreement in participants’ classification according to the fifths of the distribution of scores and the fifths of the dietary share of UPF and an inverse association between age and the relatively high frequency of UPF consumption (upper fifth of the distribution) for both score and dietary share of UPF.

Despite being conducted with a convenience sample, the distribution of participants’ consumption frequency of UPF subgroups found in our study was quite similar to that estimated by the VIGITEL system for the adult Brazilian population of the capitals of the 27 units of the Federation^13^. In both scenarios, the three subgroups more frequently consumed on the day prior to the interview were margarine (38.0% in our study and 42.6% in VIGITEL’s), loaf bread and similar (33.0% and 32.8%), and soft drinks (30.7% and 27.7%). The score distribution in the convenience sample was also similar to that observed in the VIGITEL sample, with the upper fifth of the score distribution containing scores ≥ 5 in both cases^13^.

Added to the findings reported by a similar study on the version of the score used by the VIGITEL system^14^, our results indicate the feasibility in monitoring the participation of UPF in the dietary intake in an effective, quick, and practical manner. In Brazil and in several countries worldwide, such participation has been associated with the sharp deterioration in diet quality^3^ and the increased risk of obesity, diabetes, cardiovascular diseases, and several other chronic non-communicable diseases of great epidemiological relevance^4^. As part of the Innovative Methods and Metrics for Agriculture and Nutrition Actions program (IMMANA – based at the London School of Hygiene and Tropical Medicine^c^), the Nova screener for the consumption of ultra-processed foods is being adapted for use in India, Senegal, and Ecuador, which will enable other countries to study the performance of the Nova score.

The main limitation of this study is the impossibility of extrapolating its results to Brazilian populations with low education level, since four fifths of the participants had completed at least the secondary education. We also did not evaluate the scores of men and women and in specific age groups, representing another limitation. Our sample size (n = 300) was adequate to identify even weak correlations between two methods classifications^21^, but it did not allow analyses stratification according to sociodemographic strata. Scores performance, according to gender, age, and education level, will be soon evaluated based on data collected from a subsample by quotas of the NutriNet Brasil cohort (n = 900), which monitors more than 90,000 people from all regions of the country^d^.

## CONCLUSION

The Nova score for the consumption of ultra-processed foods, obtained in a quick and practical manner using an electronic self-report questionnaire, shows a good potential in reflecting the dietary share of this food group in Brazil.

## Footnotes

a Epicollect5 [software]. Centre for Genomic Pathogen Surveillance; 2019 [cited 7 feb 2021]. Available at: https://five.epicollect.net/

b Abramson JH. WinPepiSetup.exe. Version 11.65. London, UK: Brixton Health; 2016 [cited 07 feb 2021]. Available at: http://www.brixtonhealth.com/pepi4windows.html

c Innovative Methods and Metrics for Agriculture and Nutrition Actions (IMMANA) [Internet]. Agriculture and Nutrition Actions (ANH) Academy; 2020. [cited feb. 7, 2021]. Available at: https://www.anh-academy.org/immana

d Universidade de São Paulo, Faculdade de Saúde Pública, Núcleo de Pesquisas Epidemiológicas em Nutrição e Saúde. NutriNet Brasil. São Paulo: Nupens; c2018-2021 [cited 7 feb 2021]. Available at:https://nutrinetbrasil.fsp.usp.br
