# Supplementary material for: Nova score for the consumption of ultra-processed foods: description and performance evaluation in Brazil
Source: Rev Saude Publica. 2021 Apr 5;55:13. doi: 10.11606/s1518-8787.2021055003588 (PMC8023324; doi:10.11606/s1518-8787.2021055003588)
Supplement: Translation

# Translation: Escore Nova de consumo de alimentos ultraprocessados: descrição e avaliação de desempenho no Brasil

**Authors:** Caroline dos Santos Costa, Franciane Rocha de Faria, Kamila Tiemann Gabe, Isabela Fleury Sattamini, Neha Khandpur, Fernanda Helena Marrocos Leite, Eurídice Martínez Steele, Maria Laura da Costa Louzada, Renata Bertazzi Levy, Carlos Augusto Monteiro

**Affiliations:** I Brasil Universidade de São Paulo. Núcleo de Pesquisas Epidemiológicas em Nutrição e Saúde. São Paulo, SP, Brasil; II Brasil Universidade de São Paulo. Faculdade de Saúde Pública. Programa de Pós-Graduação em Nutrição em Saúde Pública. São Paulo, SP, Brasil; III Brasil Universidade Federal de Rondonópolis. Instituto de Ciências Exatas e Naturais. Rondonópolis, MT, Brasil; IV Brasil Universidade de São Paulo. Faculdade de Saúde Pública. Departamento de Nutrição. São Paulo, SP, Brasil; V Brasil Universidade de São Paulo. Faculdade de Saúde Pública. Programa de Pós-Graduação em Saúde Global e Sustentabilidade. São Paulo, SP, Brasil; VI Brasil Universidade de São Paulo. Faculdade de Medicina. Departamento de Medicina Preventiva. São Paulo, SP, Brasil

**Keywords:** Consumo de Alimentos, Alimentos Ultraprocessados, Inquéritos sobre Dietas, métodos, Inquéritos e Questionários, Estudo de Validação

## Abstract

**OBJETIVO:**

Descrever o escore Nova de consumo de alimentos ultraprocessados e avaliar seu potencial para refletir, no contexto brasileiro, a participação desses alimentos na dieta.

**MÉTODOS:**

Estudo realizado na cidade de São Paulo com amostra de conveniência de 300 adultos, que responderam, em cerca de três minutos, em um *tablet*, a um questionário eletrônico de autorrelato sobre o consumo, no dia anterior, de 23 subgrupos de alimentos ultraprocessados comumente consumidos no Brasil. O escore de cada participante correspondeu ao número de subgrupos reportados. A participação de alimentos ultraprocessados no consumo alimentar do mesmo dia, expressa como percentual da ingestão total de energia, foi calculada por meio das respostas dos participantes a recordatório alimentar completo de 24 horas aplicado em cerca de 30 minutos por nutricionistas treinados. A associação entre o escore e a participação de ultraprocessados na dieta foi estudada por modelos de regressão linear. A concordância na classificação dos participantes segundo quintos do escore e quintos da participação de alimentos ultraprocessados na dieta foi avaliada pelo índice Pabak.

**RESULTADOS:**

O percentual médio de participação de alimentos ultraprocessados na dieta aumentou linear e significativamente com o aumento do escore Nova de consumo de alimentos ultraprocessados. Observou-se concordância substancial na classificação dos participantes segundo quintos da distribuição do escore e quintos da distribuição do percentual de participação de alimentos ultraprocessados na dieta (índice Pabak = 0,67). Relação inversa da idade com a frequência de consumo relativamente elevado de alimentos ultraprocessados (quinto superior da distribuição) foi observada tanto para o escore quanto para a participação de alimentos ultraprocessados na dieta.

**CONCLUSÃO:**

O escore Nova de consumo de alimentos ultraprocessados, obtido de forma rápida e prática, apresenta bom potencial para refletir, no contexto brasileiro, a participação desses alimentos na dieta.

## INTRODUÇÃO

Alimentos ultraprocessados, conforme definidos pelo sistema Nova de classificação de alimentos^1^, são formulações industriais de substâncias derivadas de alimentos com pouco ou nenhum alimento inteiro e frequentemente adicionadas de corantes, flavorizantes, emulsificantes, espessantes e outros aditivos cosméticos para que se tornem palatáveis ou mesmo hiperpalatáveis. Os ingredientes e processos utilizados na fabricação desses alimentos são, em sua maioria, de uso exclusivo da indústria de alimentos^2^.

Inquéritos nutricionais realizados em amostras probabilísticas da população de vários países evidenciam que o consumo de alimentos ultraprocessados, medido pelo percentual da ingestão total de energia relativa a esses alimentos, está forte e inversamente relacionado à qualidade nutricional da dieta^3^. Revisões sistemáticas de estudos de coorte bem conduzidos e de grande porte mostram que o mesmo percentual calórico proveniente de alimentos ultraprocessados está diretamente associado ao risco de doenças crônicas não transmissíveis, como obesidade, diabetes, hipertensão, dislipidemias, doenças cardio e cerebrovasculares, câncer em geral, câncer de mama e depressão, assim como à mortalidade precoce por qualquer causa^4^.

Nos estudos sobre a relação com a qualidade nutricional da dieta ou o risco de doenças crônicas, a contribuição dietética de alimentos ultraprocessados é mensurada pelo emprego de instrumentos de coleta de dados que demandam entrevistadores experientes e grande quantidade de tempo e disposição dos entrevistados, como no caso dos recordatórios alimentares de 24 horas, ou participantes com suficiente escolaridade, disposição e tempo, como no caso dos registros alimentares ou dos questionários de frequência de consumo de alimentos^11^. A complexidade desses instrumentos de coleta de dados tem impedido que o consumo de alimentos ultraprocessados seja conhecido em muitas populações e, mais ainda, que ele possa ser monitorado. Com isso, há prejuízos evidentes para a formulação e a avaliação de políticas públicas que visem reduzir o consumo de alimentos ultraprocessados.

Objetivando monitorar o consumo de alimentos ultraprocessados na população adulta brasileira acompanhada por meio de entrevistas telefônicas pelo Sistema de Vigilância de Fatores de Risco e Proteção para as Doenças Crônicas por Inquérito Telefônico (Vigitel) nas capitais das 27 unidades da federação, autores do presente estudo desenvolveram um instrumento simplificado contendo questões sobre o consumo no dia anterior (respondidas com “sim” ou “não”) de uma lista de treze subgrupos de alimentos ultraprocessados. O instrumento, inserido no questionário anual do Vigitel desde 2018^12^, permite o cálculo de um escore de consumo de alimentos ultraprocessados, que vem a ser o número de subgrupos consumidos no dia anterior pelos entrevistados, variando, portanto, entre zero e treze^13^. Esse escore mostrou boa concordância com a contribuição dietética de alimentos ultraprocessados em um estudo de validação realizado com uma amostra de conveniência com 150 participantes^14^.

Um segundo instrumento simplificado para avaliar o consumo de alimentos ultraprocessados, neste caso desenhado para ser respondido sem a participação de entrevistadores, em telefones celulares, *tablets* ou computadores, foi desenvolvido para o estudo de coorte NutriNet Brasil^15^. Este instrumento, doravante denominado rastreador Nova de consumo de alimentos ultraprocessados, gera o escore Nova de consumo de alimentos ultraprocessados. A descrição desse novo escore e uma avaliação de sua capacidade para refletir, no contexto brasileiro, a participação de alimentos ultraprocessados na dieta são apresentadas a seguir.

## MÉTODOS

### Amostra

Este estudo foi realizado na cidade de São Paulo com uma amostra de conveniência de 300 adultos com 18 ou mais anos de idade. Os participantes incluíram usuários de dois centros de saúde da Universidade de São Paulo e funcionários e alunos dessa mesma universidade.

### Coleta de Dados

A coleta de dados foi feita entre setembro e novembro de 2019 por dois nutricionistas treinados por um dos autores deste artigo (CSC). De início, os participantes foram informados do objetivo do estudo e convidados a participar. Após sua concordância e assinatura de termo de consentimento, informaram seu sexo, idade e nível de escolaridade. A seguir, responderam na tela de um *tablet*, sem ajuda do nutricionista, ao rastreador Nova de consumo de alimentos ultraprocessados, marcando, a partir de uma lista apresentada, todos os itens que haviam sido consumidos no dia anterior (formato *checkbox*). O tempo médio despendido para concluir as respostas foi de três minutos. Na sequência, inquiridos pelo nutricionista, os participantes responderam a um recordatório alimentar de 24 horas informando todos os alimentos que haviam consumido no dia anterior e as quantidades consumidas. O tempo médio para conclusão do recordatório alimentar foi de 30 minutos.

### Rastreador Nova de Consumo de Alimentos Ultraprocessados

Como no caso do instrumento utilizado no sistema Vigitel, o rastreador Nova de consumo de alimentos ultraprocessados foi construído de modo a incluir os subgrupos de alimentos ultraprocessados com maior participação na ingestão diária de energia estimada pelo inquérito nacional de consumo alimentar realizado na Pesquisa de Orçamentos Familiares (POF) do IBGE de 2008–2009^16^. Com o desdobramento de alguns dos 13 subgrupos do instrumento original, o rastreador Nova apresenta uma lista de 23 subgrupos de alimentos ultraprocessados. As questões sobre o consumo de cada um desses 23 subgrupos são apresentadas em três telas: a primeira, relativa a bebidas (seis subgrupos); a segunda, relativa a produtos que substituem ou acompanham refeições (dez subgrupos); e a terceira, relativa a produtos comumente consumidos na forma de lanches ou *snacks* (sete subgrupos), conforme exposto na Figura 1. As questões foram inseridas no *tablet* com o auxílio do software *Epicollect5 Data Collection*^a^, que armazena as respostas dos participantes na forma de banco de dados.

**Figura 1:** Rastreador Nova para avaliação do consumo de alimentos ultraprocessados na plataforma *Epicollect5 Data Collection*®.

### Recordatório Alimentar de 24 horas (R24h)

O R24h, aplicado pelos nutricionistas, empregou o método *multiple-pass*^17^. Esse método consiste em cinco etapas, começando pelo relato rápido do participante de todos os alimentos e bebidas consumidos de maneira ininterrupta, sendo o entrevistado questionado, ao final do relato rápido, se recorda mais algum outro alimento ou bebida que não tenha sido relatado, com listagem de alimentos comumente esquecidos por parte do entrevistador. Ao término desse relato, o participante é questionado sobre tipo, horário e local de cada refeição. Então, é solicitado um detalhamento dos alimentos com profundidade, incluindo, por exemplo, modo de preparo, procedência, quantidades, medidas caseiras e tamanhos, bem como informações sobre adição de outros alimentos (por exemplo, açúcar). A entrevista é finalizada com uma revisão completa dos alimentos, com listagem do relato por parte do entrevistador ao entrevistado, estimulando o relato de alimentos possivelmente esquecidos e/ou omitidos.

O projeto deste estudo foi aprovado pelo Comitê de Ética em Pesquisa da Faculdade de Saúde Pública da Universidade de São Paulo (CAAE: 83221317.5.0000.5421; número do parecer: 2.517.894) e todos os participantes assinaram o Termo de Consentimento Livre e Esclarecido.

### Análise de Dados

O escore Nova de consumo de alimentos ultraprocessados de cada participante foi calculado a partir da somatória de subgrupos de alimentos ultraprocessados reportados dentre os 23 listados, podendo variar, portanto, de 0 a 23. Para o cálculo da contribuição de alimentos ultraprocessados no consumo alimentar do dia anterior, cada um dos itens de consumo alimentar informado pelo participante no R24h foi inicialmente classificado em ultraprocessado ou não ultraprocessado de acordo com a classificação Nova^1,2^. A seguir, a quantidade de cada item de consumo informada em medidas caseiras foi transformada em gramas e convertida em calorias, utilizando-se a Tabela de Composição de Alimentos Consumidos no Brasil^18^. Por fim, foram calculados o total de calorias consumidas, as calorias provenientes de alimentos ultraprocessados e o percentual do total de calorias que eram provenientes de alimentos ultraprocessados.

Inicialmente, avaliou-se a associação entre o escore Nova de consumo de alimentos ultraprocessados e a participação de alimentos ultraprocessados na dieta. Para tanto, examinou-se a variação do percentual médio de calorias provenientes de alimentos ultraprocessados conforme a variação do escore, expresso de forma contínua e também em intervalos correspondentes a quintos de sua distribuição. Em ambos os casos, modelos de regressão linear foram empregados para testar a presença de tendência linear. Em seguida, avaliou-se o grau de concordância na classificação dos participantes segundo quintos da distribuição do percentual de calorias de alimentos ultraprocessados e segundo quintos da distribuição do escore Nova de consumo de alimentos ultraprocessados. Nessa avaliação, calculou-se o índice Pabak (*Prevalence-adjusted and bias-adjusted kappa*), que corresponde a um kappa ajustado para prevalência e viés^19^. O índice indica concordância quase perfeita quando é superior a 0,80, substancial quando fica entre 0,61 e 0,80, moderada entre 0,41 e 0,60, razoável entre 0,21 e 0,40 e fraca quando é igual ou inferior a 0,20^20^. Por fim, comparou-se a variação, segundo faixa etária, de prevalências de consumo relativamente elevado de alimentos ultraprocessados definidas por meio de dois critérios alternativos: consumo equivalente ao observado no quinto superior da distribuição do escore Nova e consumo equivalente ao observado no quinto superior da distribuição do percentual de calorias provenientes de alimentos ultraprocessados.

As análises foram realizadas com o auxílio do *software* STATA^®^ versão 16.1, e o cálculo do índice Pabak foi feito pelo *software* WINPEPI (PEPI-for-Windows) versão 11.65^b^.

## RESULTADOS

Do total de 300 voluntários entrevistados, a maioria era do sexo feminino (71,3%), tinha entre 25 e 59 anos de idade (72,3%) e havia completado o Ensino Médio ou iniciado ou completado o Ensino Superior (80,7%), como pode ser observado na Tabela 1. Já a Tabela 2 descreve a frequência de consumo no dia anterior à entrevista de cada um dos 23 subgrupos de alimentos incluídos no rastreador Nova de consumo de alimentos ultraprocessados. Cerca de um em cada três participantes referiram o consumo de margarina (38,0%), de pão de forma, cachorro-quente ou de hambúrguer (33,0%) e de refrigerante tradicional ou diet (30,7%). Entre 15% e 20% referiram o consumo de biscoito doce com ou sem recheio (19,7%), salgadinho de pacote, batata palha ou biscoito salgado (16,3%) e chocolate em barra ou bombom (15,0%). Os demais subgrupos de alimentos ultraprocessados foram consumidos no dia anterior por menos de 15% dos entrevistados.

**Tabela 1:** Distribuição segundo variáveis sociodemográficas de adultos usuários de centros de saúde, funcionários e alunos da Universidade de São Paulo (n = 300). São Paulo, Brasil, 2019.

Variáveis	N	%
Sexo		
Masculino	86	28,7
Feminino	214	71,3
Idade (anos)		
18–24	34	11,4
25–34	79	26,3
35–44	71	23,7
45–59	67	22,3
60+	49	16,3
Escolaridade		
Menos do que ensino médio completo	58	19,3
Ensino médio completo	114	38,0
Ensino superior incompleto ou completo	128	42,7

**Tabela 2:** Frequência (%) de consumo, no dia anterior à entrevista, de alimentos incluídos no rastreador Nova de consumo de alimentos ultraprocessados. Adultos usuários de centros de saúde, funcionários e alunos da Universidade de São Paulo (n = 300). São Paulo, Brasil, 2019.

Alimentos	%
Margarina	38,0
Pão de forma, cachorro-quente ou de hambúrguer	33,0
Refrigerante tradicional ou diet	30,7
Biscoito doce com ou sem recheio	19,7
Salgadinho de pacote, batata palha ou biscoito salgado	16,3
Chocolate em barra ou bombom	15,0
Presunto, salame ou mortadela	14,7
Salsicha, hambúrguer ou nuggets	13,3
Iogurte com sabor de fruta ou chocolate	12,7
Suco de fruta em caixa ou lata tipo Del Valle	12,7
Refresco em pó tipo Tang	12,0
Maionese, ketchup ou mostarda	11,7
Sorvete ou picolé de marca	10,3
Bebida achocolatada tipo Nescau	8,3
Batata frita congelada ou de redes como McDonald’s	5,3
Macarrão instantâneo tipo Miojo ou sopa de pacote	5,3
Bebida à base de chá tipo *ice tea*	4,0
Pizza congelada ou de redes como Pizza Hut ou Domino’s	3,7
Lasanha congelada ou outro prato pronto comprado congelado	3,3
Molho pronto para salada	3,0
Bolinho de pacote	2,7
Barra de cereal	2,7
Cereal matinal açucarado tipo Sucrilhos	1,7

A Tabela 3 descreve a distribuição do escore Nova de consumo de alimentos ultraprocessados (número de subgrupos consumidos no dia anterior à entrevista). O escore variou entre 0 e 9, sendo mais frequentes os escores 1, 2, 3 e 4 (19,7%, 20,3%, 19,3% e 14,0%, respectivamente). Escores nulos e escores iguais ou superiores a 5 foram observados em 9,0% e 17,7% dos participantes, respectivamente. A mesma Tabela 3 mostra que o percentual médio de participação de alimentos ultraprocessados no valor calórico total da dieta, calculado com base no R24h, aumenta linear e significativamente com o aumento do escore de consumo de alimentos ultraprocessados. A Tabela 4 mostra que a distribuição dos participantes levando em conta sua classificação simultânea segundo quintos da participação de alimentos ultraprocessados na dieta, calculada pelo R24h, e segundo quintos aproximados do escore Nova (0–1, 2, 3, 4 e ≥ 5) indica concordância substancial entre os dois critérios (índice Pabak de 0,67).

**Tabela 3:** Participação de alimentos ultraprocessados na dieta, calculada pelo recordatório alimentar de 24h, segundo o escore Nova de consumo de alimentos ultraprocessados. Adultos usuários de centros de saúde, funcionários e alunos da Universidade de São Paulo (n=300). São Paulo, Brasil, 2019.

Escore Nova de consumo de alimentos ultraprocessados	Amostra n (%)	Participação de alimentos ultraprocessados na dieta (% da energia total) Média (IC95%)
0	27 (9,0)	9,4 (2,3–16,6)
1	59 (19,7)	23,8 (19,0–28,6)
2	61 (20,3)	31,6 (26,9–36,4)
3	58 (19,3)	31,1 (26,2–35,9)
4	42 (14,0)	35,6 (29,8–41,3)
5	23 (7,7)	37,5 (29,8–45,2)
6	17 (5,7)	54,1 (45,2–63,1)
7	8 (2,7)	47,0 (34,0–60,1)
8	1 (0,3)	27,5 (-9,4–64,5)
9	4 (1,3)	35,6 (17,1–54,0)^a^
0–1	86 (28,7)	19,3 (15,2–23,4)
2	61 (20,3)	31,6 (26,8–36,5)
3	58 (19,3)	31,1 (26,1–36,1)
4	42 (14,0)	35,6 (29,7–41,4)
5 ou +	53 (17,7)	43,9 (38,7–49,1)^a^

IC95%: intervalo de confiança de 95%

^a^ Valor-p para tendência linear < 0,001.

**Tabela 4:** Distribuição (%) segundo quintos da participação de alimentos ultraprocessados na dieta e quintos (aproximados) do escore Nova de consumo de alimentos ultraprocessados. Adultos usuários de centros de saúde, funcionários e alunos da Universidade de São Paulo (n = 300). São Paulo, Brasil, 2019.

Quintos do escore Nova de consumo de ultraprocessados	Quintos da participação de ultraprocessados na dieta (% do total de calorias)	0–1	2	3	4	5 ou +	Total
Q1 (≤ 11,0)		13,0	2,0	2,3	2,0	0,7	20,0
Q2 (11,1–20,4)		6,0	5,0	4,0	2,7	2,3	20,0
Q3 (20,5–34,8)		4,0	4,7	6,0	2,3	3,0	20,0
Q4 (34,9–49,5)		3,3	5,0	3,7	3,0	5,0	20,0
Q5 (≥ 49,6)		2,3	3,7	3,3	4,0	6,7	20,0
Total		28,6	20,4	19,3	14,0	17,7	100,0

Nota: Índice Pabak (kappa ajustado para prevalência e viés) = 0,67.

A Figura 2 apresenta a variação com a faixa etária dos participantes na prevalência de consumo relativamente alto de alimentos ultraprocessados, adotando-se como critério de consumo elevado o consumo observado, alternativamente, no quinto superior (aproximado) da distribuição do escore Nova de consumo de alimentos ultraprocessados (≥ 5) e no quinto superior da distribuição da participação de alimentos ultraprocessados no valor calórico total da dieta (≥ 49,6% do total de calorias). Com os dois critérios, observa-se que a prevalência de consumo relativamente elevado de alimentos ultraprocessados diminui linearmente com o aumento da idade (p = 0,038 e p = 0,001, respectivamente).

**Figura 2:** Variação na prevalência (%) de alto consumo de alimentos ultraprocessados segundo faixa etária conforme dois critérios. Adultos usuários de centros de saúde, funcionários e alunos da Universidade de São Paulo (n = 300). São Paulo, Brasil, 2019.

^a^ Valor-p = 0,038 para tendência linear.

^b^ Valor-p = 0,001 para tendência linear.

## DISCUSSÃO

Os resultados do presente estudo, realizado com uma amostra de conveniência recrutada na cidade de São Paulo, no Brasil, evidenciaram que o escore Nova de consumo de alimentos ultraprocessados, obtido com um questionário eletrônico autorrespondível em cerca de três minutos, mostrou-se direta e linearmente associado ao percentual da ingestão total de energia proveniente de alimentos ultraprocessados, obtido com recordatório alimentar de 24 horas aplicado em cerca de 30 minutos por nutricionista treinado. Evidenciaram também concordância substancial na classificação dos participantes segundo quintos da distribuição do escore e quintos da distribuição do percentual de participação de alimentos ultraprocessados na dieta. Adicionalmente, uma clara relação inversa da idade com a frequência de consumo relativamente elevado de alimentos ultraprocessados (quinto superior da distribuição) foi observada tanto com o escore quanto com a participação de alimentos ultraprocessados na dieta.

Embora o presente estudo tenha sido realizado em uma amostra de conveniência, a distribuição da frequência de consumo de subgrupos de alimentos ultraprocessados pelos participantes foi bastante semelhante à estimada pelo sistema Vigitel para a população brasileira adulta das capitais das 27 unidades da federação^13^. Por exemplo, nas duas situações, os três subgrupos com maior frequência de consumo no dia anterior foram os mesmos: margarina (38,0% e 42,6%, neste estudo e no Vigitel, respectivamente), pão de forma e assemelhados (33,0% e 32,8%) e refrigerantes (30,7% e 27,7%). A distribuição do escore na amostra de conveniência foi também semelhante à observada na amostra do Vigitel, com o quinto superior da distribuição do escore incluindo escores ≥ 5 nos dois casos^13^.

Os achados deste estudo, somados aos de estudo similar sobre a versão do escore utilizada pelo sistema Vigitel^14^, indicam ser possível monitorar a participação de alimentos ultraprocessados na dieta de forma eficaz, rápida e prática. Note-se que essa participação vem sendo associada, no Brasil e em vários países do mundo, à intensa deterioração da qualidade das dietas^3^ e ao risco aumentado de obesidade, diabetes, doenças cardiovasculares e várias outras doenças crônicas não transmissíveis de grande relevância epidemiológica^4^. Como parte do programa Immana (*The Innovative Methods and Metrics for Agriculture and Nutrition Actions*), baseado na *London School of Hygiene and Tropical Medicine*^c^, o rastreador Nova de consumo de alimentos ultraprocessados está sendo adaptado para uso na Índia, Senegal e Equador, o que permitirá que o desempenho do escore Nova de consumo de alimentos ultraprocessados seja estudado em outros países.

A principal limitação deste estudo se refere à impossibilidade de extrapolar seus resultados para populações brasileiras com baixo nível de escolaridade, uma vez que quatro quintos dos participantes haviam completado pelo menos o curso médio. Outra limitação importante foi a não avaliação do desempenho do escore em homens e mulheres e em faixas etárias específicas. Embora o número de participantes no presente estudo (300) seja adequado para identificar mesmo correlações fracas entre classificações feitas por dois métodos^21^, ele não permitiu estratificação das análises segundo estratos sociodemográficos. O desempenho do escore segundo sexo, idade e nível de escolaridade das pessoas será avaliado brevemente com base em dados já coletados de uma subamostra por cotas da coorte NutriNet Brasil (n = 900), que já acompanha mais de 90 mil pessoas de todas as regiões do país^d^.

## CONCLUSÃO

O escore Nova de consumo de alimentos ultraprocessados, obtido de forma rápida e prática por questionário eletrônico de autorrelato, apresenta bom potencial para refletir, no contexto brasileiro, a participação desses alimentos na dieta.

## Footnotes

a Epicollect5 [software]. Centre for Genomic Pathogen Surveillance; 2019 [citado 7 fev 2021]. Disponível em: https://five.epicollect.net/

b Abramson JH. WinPepiSetup.exe. Version 11.65. London (UK): Brixton Health; 2016 [citado 07 fev 2021]. Disponível em: http://www.brixtonhealth.com/pepi4windows.html

c Innovative Methods and Metrics for Agriculture and Nutrition Actions (IMMANA). Agriculture and Nutrition Actions (ANH) Academy; 2020. [citado 7 fev 2021]. Disponível em: https://www.anh-academy.org/immana

d Universidade de São Paulo, Faculdade de Saúde Pública, Núcleo de Pesquisas Epidemiológicas em Nutrição e Saúde. NutriNet Brasil. São Paulo: Nupens; c2018-2021 [citado 7 fev 2021]. Disponível em: https://nutrinetbrasil.fsp.usp.br/
